# Life Cycle Inventory datasets for nano-grid configurations

**DOI:** 10.1016/j.dib.2019.104895

**Published:** 2019-11-28

**Authors:** Federico Rossi, Maria Laura Parisi, Simone Maranghi, Riccardo Basosi, Adalgisa Sinicropi

**Affiliations:** a University of Siena, R^2^ES Lab, Department of Biotechnology, Chemistry and Pharmacy, Via A. Moro,2, Siena, Italy; b University of Florence, Department of Industrial Engineering, Via Santa Marta,3, Florence, Italy; c CSGI, Center for Colloid and Surface Science, Via Della Lastruccia 3, 50019, Sesto Fiorentino, Italy; d National Research Council, Institute of Chemistry of Organometallic Compounds (CNR-ICCOM), Via Madonna Del Piano 10, 50019 Sesto Fiorentino, Italy

**Keywords:** Storage, Photovoltaics, Smart grids, Life cycle assessment, Batteries, Solar energy

## Abstract

Datasets concerning some user-scale Smart Grids, named Nano-grids, are reported in this paper. First several Solar Home Systems composed of a photovoltaic plant, a backup generator and different types of lithium-ion batteries are provided. Then, the inventory analysis of hybrid Nano-grids integrating batteries and hydrogen storage is outlined according to different scenarios. These data inventory could be useful for any academic or stakeholder interested in reproducing this analysis and/or developing environmental sustainability assessment in the field of Smart Grids. For more insight, please see “Environmental analysis of a Nano-Grid: a Life Cycle Assessment” by Rossi F, Parisi M.L., Maranghi S., Basosi R., Sinicropi A. [1].

**Table:** Specifications Table

Subject	Renewable Energy, Sustainability and the Environment
Specific subject area	Life Cycle Assessment
Type of data	Tables
How data were acquired	Ecoinvent 3.2 database and scientific literature
Data format	Raw Analyzed
Parameters for data collection	Technological, temporal and geographical representativeness of data are described in Ecoinvent 3.2 reports.
Description of data collection	Data collection is performed employing the Ecoinvent 3.2 database. When the required information is not available from the Ecoinvent database, secondary data are acquired from literature.
Data source location	Institution: Ecoinvent City/Town/Region: Zurich Country: Switzerland
Data accessibility	The Life Cycle Inventories are reported with this article
Related research article	Federico Rossi, Maria Laura Parisi, Simone Maranghi, Riccardo Basosi, Adalgisa Sinicropi “Environmental analysis of a Nano-Grid: a Life Cycle Assessment” (https://doi.org/10.1016/j.scitotenv.2019.134814)

**Table:** 

**Value of the Data** • Life Cycle Inventories for nano-grids components and manufacturing processes concerning raw materials and energy input-output flows are provided. • Data are useful for any academics studying smart grids value chain and for any stakeholders interested in the environmental sustainability of energy systems network. • These comprehensive life cycle inventories can be employed for direct use or as data-proxies to be further customized and adapted for the development of environmental Life Cycle Assessment studies in the field of Smart Grids. • Up-to-date datasets are built from technical data presented in scientific reports and papers and modelled according to the Ecoinvent 3.2 database for easy employment and reproducibility

## 1 Data

Datasets concerning some user-scale Smart Grids (Nano-grids) components and manufacturing processes are presented. Several Solar Home Systems composed of a photovoltaic plant, a backup generator and different types of lithium-ion batteries are described. Then, the inventory analysis of hybrid Nano-grids integrating batteries and hydrogen storage is outlined according to different scenarios [1]. The inventory analysis presented in this paper corresponds to the Life Cycle Inventory (LCI) phase that is a mandatory phase of the Life Cycle Assessment methodology, ISO 14040 standardized procedure for the environmental impact analysis of a product or a system. The LCI consists of a comprehensive dataset containing quantitative information about all the energy and matter flows involved in the life cycle of a product, process or system. The inventory analysis is performed using openLCA and is based on the Ecoinvent 3.2 database. In case some of the components of the Nano-grids are not present in the database, secondary data must be recovered from the literature, in order to create a representative Life Cycle Inventory for the missing components [[2], [3], [4]]. Table 1, Table 2 describe the LCI of two different types of Hydrogen Storage Tanks during the production phase. Tables from Table 3, Table 4, Table 5, Table 6, Table 7, Table 8, Table 9 summarize the LCI of the Solar Home Systems whereas tables from Table 10, Table 11, Table 12, Table 13 describe the LCIs of hybrid Nano-grids with four different scenarios [1]. Concerning tables from Table 14, Table 15, Table 16, Table 17, Table 18, Table 19, they represent the inventories dealing with the end of life of lithium-ion batteries, photovoltaic panels, electricity converters, proton exchange membrane fuel cells and electrolysers and hydrogen storage tanks.

**Table 1:** Life Cycle Inventory of Type III Hydrogen Storage Tank production [6].

Component	Amount	Unit	Process	Comments and Sources
*Input*				
carbon fibre	21.2	kg	market for ammonia, liquid | ammonia, liquid | APOS, U - RER	Carbon fibre production [7]
	404.9	MJ	market for electricity, low voltage | electricity, low voltage | APOS, U - IT	Carbon fibre production [7]
	53.0	kg	market for propylene | propylene | APOS, U - GLO	Carbon fibre production [7]
chromium steel pipe	4.0	kg	chromium steel pipe production | chromium steel pipe | APOS, U - GLO	Ecoinvent 3.2 [8]
glass fibre reinforced plastic, polyester resin, hand lay-up	6.1	kg	market for glass fibre reinforced plastic, polyester resin, hand lay-up| glass fibre reinforced plastic, polyester resin, hand lay-up | APOS, U - GLO	Ecoinvent 3.2 [8]
polyethylene, high density, granulate	11.4	kg	market for polyethylene, high density, granulate | polyethylene, high density, granulate | APOS, U - GLO	Ecoinvent 3.2 [8] provides HDPE at granulate grade.
polymer foaming	5.2	kg	market for polymer foaming | polymer foaming | APOS, U - GLO	Ecoinvent 3.2 [8]
silicon, electronics grade	1.0	kg	market for silicon, electronics grade | silicon, electronics grade | APOS, U - GLO	Ecoinvent 3.2 [8]
steel, low-alloyed	14.5	kg	market for steel, low-alloyed | steel, low-alloyed | APOS, U - GLO	It contains all the steel-based parts [8].
*Output*				
Type III Hydrogen storage tank	258.0	l	Hydrogen storage tank production 350 bar	Reference output

**Table 2:** Life Cycle Inventory of Type IV Hydrogen Storage Tank production [6].

Component	Amount	Unit	Process	Comments and Sources
*Input*				
carbon fibre	27.0	kg	market for ammonia, liquid | ammonia, liquid | APOS, U - RER	Carbon fibre production [7]
	514.9	kg	market for electricity, low voltage | electricity, low voltage | APOS, U - IT	Carbon fibre production [7]
	67.4	kg	market for propylene | propylene | APOS, U - GLO	Carbon fibre production [7]
chromium steel pipe	4	kg	chromium steel pipe production | chromium steel pipe | APOS, U - GLO	Ecoinvent 3.2 [8]
glass fibre reinforced plastic, polyester resin, hand lay-up	4.6	kg	market for glass fibre reinforced plastic, polyester resin, hand lay-up| glass fibre reinforced plastic, polyester resin, hand lay-up | APOS, U - GLO	Ecoinvent 3.2 [8]
polyethylene, high density, granulate	8.0	kg	market for polyethylene, high density, granulate | polyethylene, high density, granulate | APOS, U - GLO	Ecoinvent 3.2 [8] provides HDPE at granulate grade.
polymer foaming	4.0	kg	market for polymer foaming | polymer foaming | APOS, U - GLO	Ecoinvent 3.2 [8]
silicon, electronics grade	1.0	kg	market for silicon, electronics grade | silicon, electronics grade | APOS, U - GLO	Ecoinvent 3.2 [8]
steel, low-alloyed	13.7	kg	market for steel, low-alloyed | steel, low-alloyed | APOS, U - GLO	It contains all the steel-based parts [8].
*Output*				
Type IV Hydrogen storage tank	149.0	l	Hydrogen storage tank production 700 bar	Reference output

**Table 3:** Life Cycle Inventory of a SHS equipped with M-B (LFP) LIBs.

Component	Amount	Unit	Process	Comments and Sources
*Input*				
PV panels	34.9	m^2^	market for photovoltaic panel, single-Si wafer | photovoltaic panel, single-Si wafer | APOS, U - GLO	Ecoinvent 3.2 [8]
Is	2.5	Items	market for inverter, 2.5kW | inverter, 2.5kW | APOS, U - GLO	Ecoinvent 3.2 [8]
CCs	27.5	kg	charger production, for electric passenger car | charger, electric passenger car | APOS, U - GLO	Ecoinvent 3.2 [8]
Wiring	3.5	kg	cable production, unspecified | cable, unspecified | APOS, U - GLO	Evaluation based on [11]
	2.1	kg	tube insulation production, elastomere | tube insulation, elastomere | APOS, U - DE	Evaluation based on [11]
Backup Energy	15.1	MWh	market for diesel, burned in diesel-electric generating set, 18.5kW | diesel, burned in diesel-electric generating set, 18.5kW | APOS, U - GLO	Ecoinvent 3.2 [8]
M-B (LFP) LIBs	438.2	kg	Li-Ion battery pack production, LFP-C, modular, at plant (NTNU)	Database imported from Ref. [10]
*Output*				
Electricity	100.4	MWh		Reference output

**Table 4:** Life Cycle Inventory of a SHS equipped with Zack (LFP) LIBs.

Component	Amount	Unit	Process	Comments and Sources
*Input*				
PV panels	34.9	m^2^	market for photovoltaic panel, single-Si wafer | photovoltaic panel, single-Si wafer | APOS, U - GLO	Ecoinvent 3.2 [8]
Is	2.5	Items	market for inverter, 2.5kW | inverter, 2.5kW | APOS, U - GLO	Ecoinvent 3.2 [8]
CCs	27.5	kg	charger production, for electric passenger car | charger, electric passenger car | APOS, U - GLO	Ecoinvent 3.2 [8]
Wiring	3.5	kg	cable production, unspecified | cable, unspecified | APOS, U - GLO	Evaluation based on [11]
	2.1	kg	tube insulation production, elastomere | tube insulation, elastomere | APOS, U - DE	Evaluation based on [11]
Backup Energy	15.5	MWh	market for diesel, burned in diesel-electric generating set, 18.5kW | diesel, burned in diesel-electric generating set, 18.5kW | APOS, U - GLO	Ecoinvent 3.2 [8]
Zack (LFP) LIBs	753.9	kg	LFP-C type Li-Ion Battery, modular, at plant (Zackrisson, org.)	Database imported from Ref. [10]
*Output*				
Electricity	100.4	MWh		Reference output

**Table 5:** Life Cycle Inventory of a SHS equipped with Bauer (LTO) LIBs.

Component	Amount	Unit	Process	Comments and Sources
*Input*				
PV panels	34.9	m^2^	market for photovoltaic panel, single-Si wafer | photovoltaic panel, single-Si wafer | APOS, U - GLO	Ecoinvent 3.2 [8]
Is	2.5	Items	market for inverter, 2.5kW | inverter, 2.5kW | APOS, U - GLO	Ecoinvent 3.2 [8]
CCs	27.5	kg	charger production, for electric passenger car | charger, electric passenger car | APOS, U - GLO	Ecoinvent 3.2 [8]
Wiring	3.5	kg	cable production, unspecified | cable, unspecified | APOS, U - GLO	Evaluation based on [11]
	2.1	kg	tube insulation production, elastomere | tube insulation, elastomere | APOS, U - DE	Evaluation based on [11]
Backup Energy	14.9		market for diesel, burned in diesel-electric generating set, 18.5kW | diesel, burned in diesel-electric generating set, 18.5kW | APOS, U - GLO	Ecoinvent 3.2 [8]
Bauer (LTO) LIBs	734.7	kg	Li-Ion Battery Pack production, LFP-TiO, modular (Bauer)	Database imported from Ref. [10]
*Output*				
Electricity	100.4	MWh		Reference output

**Table 6:** Life Cycle Inventory of a SHS equipped with Notter (LMO) LIBs.

Component	Amount	Unit	Process	Comments and Sources
*Input*				
PV panels	34.9	m^2^	market for photovoltaic panel, single-Si wafer | photovoltaic panel, single-Si wafer | APOS, U - GLO	Ecoinvent 3.2 [8]
Is	2.5	Items	market for inverter, 2.5kW | inverter, 2.5kW | APOS, U - GLO	Ecoinvent 3.2 [8]
CCs	27.5	kg	charger production, for electric passenger car | charger, electric passenger car | APOS, U - GLO	Ecoinvent 3.2 [8]
Wiring	3.5	kg	cable production, unspecified | cable, unspecified | APOS, U - GLO	Evaluation based on [11]
	2.1	kg	tube insulation production, elastomere | tube insulation, elastomere | APOS, U - DE	Evaluation based on [11]
Backup Energy	13.9	MWh	market for diesel, burned in diesel-electric generating set, 18.5kW | diesel, burned in diesel-electric generating set, 18.5kW | APOS, U - GLO	Ecoinvent 3.2 [8]
Notter (LMO) LIBs	764.9	kg	Li-ion battery, LMO-C, modular | cut-off, U (Notter/ecoinvent) - GLO	Database imported from Ref. [10]
*Output*				
Electricity	100.4	MWh		Reference output

**Table 7:** Life Cycle Inventory of a SHS equipped with Bauer (NCA) LIBs.

Component	Amount	Unit	Process	Comments and Sources
*Input*				
PV panels	34.9	m^2^	market for photovoltaic panel, single-Si wafer | photovoltaic panel, single-Si wafer | APOS, U - GLO	Ecoinvent 3.2 [8]
Is	2.5	Items	market for inverter, 2.5kW | inverter, 2.5kW | APOS, U - GLO	Ecoinvent 3.2 [8]
CCs	27.5	kg	charger production, for electric passenger car | charger, electric passenger car | APOS, U - GLO	Ecoinvent 3.2 [8]
Wiring	3.5	kg	cable production, unspecified | cable, unspecified | APOS, U - GLO	Evaluation based on [11]
	2.1	kg	tube insulation production, elastomere | tube insulation, elastomere | APOS, U - DE	Evaluation based on [11]
Backup Energy	13.9	MWh	market for diesel, burned in diesel-electric generating set, 18.5kW | diesel, burned in diesel-electric generating set, 18.5kW | APOS, U - GLO	Ecoinvent 3.2 [8]
Bauer (NCA) LIBs	259.2	kg	Li-Ion Battery Pack production, NCA-C, modular (Bauer)	Database imported from Ref. [10]
*Output*				
Electricity	100.4	MWh		Reference output

**Table 8:** Life Cycle Inventory of a SHS equipped with Ell (NCM) LIBs.

Component	Amount	Unit	Process	Comments and Sources
*Input*				
PV panels	34.9	m^2^	market for photovoltaic panel, single-Si wafer | photovoltaic panel, single-Si wafer | APOS, U - GLO	Ecoinvent 3.2 [8]
Is	2.5	Items	market for inverter, 2.5kW | inverter, 2.5kW | APOS, U - GLO	Ecoinvent 3.2 [8]
CCs	27.5	kg	charger production, for electric passenger car | charger, electric passenger car | APOS, U - GLO	Ecoinvent 3.2 [8]
Wiring	3.5	kg	cable production, unspecified | cable, unspecified | APOS, U - GLO	Evaluation based on [11]
	2.1	kg	tube insulation production, elastomere | tube insulation, elastomere | APOS, U - DE	Evaluation based on [11]
Backup Energy	13.2	MWh	market for diesel, burned in diesel-electric generating set, 18.5kW | diesel, burned in diesel-electric generating set, 18.5kW | APOS, U - GLO	Ecoinvent 3.2 [8]
Ell (NCM) LIBs	376.8	kg	Li-Ion battery pack production, NCM-C, modular (Ellingsen)	Database imported from Ref. [10]
*Output*				
Electricity	100.4	MWh		Reference output

**Table 9:** Life Cycle Inventory of a SHS equipped with M-B (NCM) LIBs.

Component	Amount	Unit	Process	Comments and Sources
*Input*				
PV panels	34.9	m^2^	market for photovoltaic panel, single-Si wafer | photovoltaic panel, single-Si wafer | APOS, U - GLO	Ecoinvent 3.2 [8]
Is	2.5	Items	market for inverter, 2.5kW | inverter, 2.5kW | APOS, U - GLO	Ecoinvent 3.2 [8]
CCs	27.5	kg	charger production, for electric passenger car | charger, electric passenger car | APOS, U - GLO	Ecoinvent 3.2 [8]
Wiring	3.5	kg	cable production, unspecified | cable, unspecified | APOS, U - GLO	Evaluation based on [11]
	2.1	kg	tube insulation production, elastomere | tube insulation, elastomere | APOS, U - DE	Evaluation based on [11]
Backup Energy	13.9	MWh	market for diesel, burned in diesel-electric generating set, 18.5kW | diesel, burned in diesel-electric generating set, 18.5kW | APOS, U - GLO	Ecoinvent 3.2 [8]
M-B (NCM) LIBs	268.9	kg	Li-Ion battery pack production, NCM-C, modular, at plant (NTNU)	Database imported from Ref. [10]
*Output*				
Electricity	100.4	MWh		Reference output

**Table 10:** Life cycle inventory of a HNG-A.

Component	Amount	Unit	Process	Comments and Sources
*Input*				
PV panels	34.9	m^2^	market for photovoltaic panel, single-Si wafer | photovoltaic panel, single-Si wafer | APOS, U - GLO	Ecoinvent 3.2 [8]
Is	2.5	Items	market for inverter, 2.5kW | inverter, 2.5kW | APOS, U - GLO	Ecoinvent 3.2 [8]
CCs	27.5	kg	charger production, for electric passenger car | charger, electric passenger car | APOS, U - GLO	Ecoinvent 3.2 [8]
Wiring	3.5	kg	cable production, unspecified | cable, unspecified | APOS, U - GLO	Evaluation based on [11]
	2.1	kg	tube insulation production, elastomere | tube insulation, elastomere | APOS, U - DE	Evaluation based on [11]
Backup Energy	0.4	MWh	market for diesel, burned in diesel-electric generating set, 18.5kW | diesel, burned in diesel-electric generating set, 18.5kW | APOS, U - GLO	Ecoinvent 3.2 [8]
Bauer (NCA) LIBs	259.2	kg	Li-Ion Battery Pack production, NCA-C, modular (Bauer)	Database imported from Ref. [10]
Type III Hydrogen storage tank	8.8	m^3^	Hydrogen storage tank production 350 bar	Table 1
Compressor	0.4	Items	air compressor production, screw-type compressor, 4kW | air compressor, screw-type compressor, 4kW | APOS, U - RER	Ecoinvent 3.2 [8]
PEMFCs	2.5	Items	fuel cell production, polymer electrolyte membrane, 2kW electrical, future | fuel cell, polymer electrolyte membrane, 2kW electrical, future | APOS, U - CH	Ecoinvent 3.2 [8]
PEMEs	2.8	Items	fuel cell production, polymer electrolyte membrane, 2kW electrical, future | fuel cell, polymer electrolyte membrane, 2kW electrical, future | APOS, U - CH	Ecoinvent 3.2 [8]
Water	10.8	m^3^	water production, deionised, from tap water, at user | water, deionised, from tap water, at user | APOS, U - CH	Ecoinvent 3.2 [8]
*Output*				
Electricity	100.4	MWh		Reference output
Compressed hydrogen	507.8	kg		By-product

**Table 11:** Life cycle inventory of a HNG-B.

Component	Amount	Unit	Process	Comments and Sources
*Input*				
PV panels	34.9	m^2^	market for photovoltaic panel, single-Si wafer | photovoltaic panel, single-Si wafer | APOS, U - GLO	Ecoinvent 3.2 [8]
Is	2.5	Items	market for inverter, 2.5kW | inverter, 2.5kW | APOS, U - GLO	Ecoinvent 3.2 [8]
CCs	27.5	kg	charger production, for electric passenger car | charger, electric passenger car | APOS, U - GLO	Ecoinvent 3.2 [8]
Wiring	3.5	kg	cable production, unspecified | cable, unspecified | APOS, U - GLO	Evaluation based on [11]
	2.1	kg	tube insulation production, elastomere | tube insulation, elastomere | APOS, U - DE	Evaluation based on [11]
Backup Energy	0.5	MWh	market for diesel, burned in diesel-electric generating set, 18.5kW | diesel, burned in diesel-electric generating set, 18.5kW | APOS, U - GLO	Ecoinvent 3.2 [8]
Bauer (NCA) LIBs	259.2	kg	Li-Ion Battery Pack production, NCA-C, modular (Bauer)	Database imported from Ref. [10]
Type IV Hydrogen storage tank	4.5	m^3^	Hydrogen storage tank production 700 bar	Table 2
Compressor	0.6	Items	air compressor production, screw-type compressor, 4kW | air compressor, screw-type compressor, 4kW | APOS, U - RER	Ecoinvent 3.2 [8]
PEMFCs	2.5	Items	fuel cell production, polymer electrolyte membrane, 2kW electrical, future | fuel cell, polymer electrolyte membrane, 2kW electrical, future | APOS, U - CH	Ecoinvent 3.2 [8]
PEMEs	2.8	Items	fuel cell production, polymer electrolyte membrane, 2kW electrical, future | fuel cell, polymer electrolyte membrane, 2kW electrical, future | APOS, U - CH	Ecoinvent 3.2 [8]
Water	10.8	m^3^	water production, deionised, from tap water, at user | water, deionised, from tap water, at user | APOS, U - CH	Ecoinvent 3.2 [8]
*Output*				
Electricity	100.4	MWh		Reference output
Compressed hydrogen	470.6	kg		By-product

**Table 12:** Life cycle inventory of a HNG-C.

Component	Amount	Unit	Process	Comments and Sources
*Input*				
PV panels	34.9	m^2^	market for photovoltaic panel, single-Si wafer | photovoltaic panel, single-Si wafer | APOS, U - GLO	Ecoinvent 3.2 [8]
Is	2.5	Items	market for inverter, 2.5kW | inverter, 2.5kW | APOS, U - GLO	Ecoinvent 3.2 [8]
CCs	27.5	kg	charger production, for electric passenger car | charger, electric passenger car | APOS, U - GLO	Ecoinvent 3.2 [8]
Wiring	3.5	kg	cable production, unspecified | cable, unspecified | APOS, U - GLO	Evaluation based on [11]
	2.1	kg	tube insulation production, elastomere | tube insulation, elastomere | APOS, U - DE	Evaluation based on [11]
Backup Energy	0.4	MWh	market for diesel, burned in diesel-electric generating set, 18.5kW | diesel, burned in diesel-electric generating set, 18.5kW | APOS, U - GLO	Ecoinvent 3.2 [8]
Bauer (NCA) LIBs	259.2	kg	Li-Ion Battery Pack production, NCA-C, modular (Bauer)	Database imported from Ref. [10]
Type III Hydrogen storage tank	8.8	m^3^	Hydrogen storage tank production 350 bar	Table 1
Compressor	0.4	Items	air compressor production, screw-type compressor, 4kW | air compressor, screw-type compressor, 4kW | APOS, U - RER	Ecoinvent 3.2 [8]
PEMFCs	0.5	Items	fuel cell production, polymer electrolyte membrane, 2kW electrical, future | fuel cell, polymer electrolyte membrane, 2kW electrical, future | APOS, U - CH	Ecoinvent 3.2 [8]
PEMEs	0.6	Items	fuel cell production, polymer electrolyte membrane, 2kW electrical, future | fuel cell, polymer electrolyte membrane, 2kW electrical, future | APOS, U - CH	Ecoinvent 3.2 [8]
Water	10.8	m^3^	water production, deionised, from tap water, at user | water, deionised, from tap water, at user | APOS, U - CH	Ecoinvent 3.2 [8]
*Output*				
Electricity	100.4	MWh		Reference output
Compressed hydrogen	507.8	kg		By-product

**Table 13:** Life cycle inventory of a HNG-D.

Component	Amount	Unit	Process	Comments and Sources
*Input*				
PV panels	34.9	m^2^	market for photovoltaic panel, single-Si wafer | photovoltaic panel, single-Si wafer | APOS, U - GLO	Ecoinvent 3.2 [8]
Is	2.5	Items	market for inverter, 2.5kW | inverter, 2.5kW | APOS, U - GLO	Ecoinvent 3.2 [8]
CCs	27.5	kg	charger production, for electric passenger car | charger, electric passenger car | APOS, U - GLO	Ecoinvent 3.2 [8]
Wiring	3.5	kg	cable production, unspecified | cable, unspecified | APOS, U - GLO	Evaluation based on [11]
	2.1	kg	tube insulation production, elastomere | tube insulation, elastomere | APOS, U - DE	Evaluation based on [11]
Backup Energy	0.5	MWh	market for diesel, burned in diesel-electric generating set, 18.5kW | diesel, burned in diesel-electric generating set, 18.5kW | APOS, U - GLO	Ecoinvent 3.2 [8]
Bauer (NCA) LIBs	259.2	kg	Li-Ion Battery Pack production, NCA-C, modular (Bauer)	Database imported from Ref. [10]
Type IV Hydrogen storage tank	4.5	m^3^	Hydrogen storage tank production 700 bar	Table 2
Compressor	0.6	Items	air compressor production, screw-type compressor, 4kW | air compressor, screw-type compressor, 4kW | APOS, U - RER	Ecoinvent 3.2 [8]
PEMFCs	0.5	Items	fuel cell production, polymer electrolyte membrane, 2kW electrical, future | fuel cell, polymer electrolyte membrane, 2kW electrical, future | APOS, U - CH	Ecoinvent 3.2 [8]
PEMEs	0.6	Items	fuel cell production, polymer electrolyte membrane, 2kW electrical, future | fuel cell, polymer electrolyte membrane, 2kW electrical, future | APOS, U - CH	Ecoinvent 3.2 [8]
Water	10.8	m^3^	water production, deionised, from tap water, at user | water, deionised, from tap water, at user | APOS, U - CH	Ecoinvent 3.2 [8]
*Output*				
Electricity	100.4	MWh		Reference output
Compressed hydrogen	470.6	kg		By-product

**Table 14:** Life Cycle Inventory of a LIBs end of life based on Ecoinvent 3.2 [8] and Weber et al. [16].

Component	Amount	Unit	Process	Comments and Sources
*Input*				
diesel, burned in building machine	0.1	MJ	diesel, burned in building machine | diesel, burned in building machine | APOS, U - GLO	Ecoinvent 3.2 [8]
electricity, medium voltage	10	Wh	electricity voltage transformation from high to medium voltage | electricity, medium voltage | APOS, U - IT	Ecoinvent 3.2 [8]
Iron scrap, sorted, pressed	0.3	kg	market for iron scrap, sorted, pressed | iron scrap, sorted, pressed | APOS, U - GLO	Ecoinvent 3.2 [8]
Used cable	−70.5	g	market for used cable | used cable | APOS, U - GLO	Ecoinvent 3.2 [8]
treatment of used Li-ion battery, hydrometallurgical treatment	−340.0	g	treatment of used Li-ion battery, hydrometallurgical treatment | used Li-ion battery | APOS, U - GLO	Ecoinvent 3.2 [8]
treatment of used Li-ion battery, pyrometallurgical treatment	−340.0	g	treatment of used Li-ion battery, pyrometallurgical treatment | used Li-ion battery | APOS, U - GLO	Ecoinvent 3.2 [8]
waste electric and electronic equipment	−31.0	g	treatment of waste electric and electronic equipment, shredding | waste electric and electronic equipment | APOS, U - GLO	Ecoinvent 3.2 [8]
waste plastic, consumer electronics	−41.0	g	treatment of waste plastic, consumer electronics, municipal incineration | waste plastic, consumer electronics | APOS, U - CH	Ecoinvent 3.2 [8]
used battery	1.0	kg		Reference input
*Output*				
Cable, unspecified	7.1	g	market for cable, unspecified | cable, unspecified | APOS, U - GLO	Avoided product Ecoinvent 3.2 [8]
Electronic scrap	31.0	g	market for electronics scrap | electronics scrap | APOS, U - GLO	Avoided product Ecoinvent 3.2 [8]
Iron scrap, sorted, pressed	26.0	g	gold-silver-zinc-lead-copper mining and beneficiation | iron scrap, sorted, pressed | APOS, U - CA-QC	Avoided product Ecoinvent 3.2 [8]

**Table 15:** Life Cycle Inventory of a PV end of life [17].

Component	Amount	Unit	Process	Comments and Sources
*Input*				
aluminium scrap, post-consumer	−182.7	kg	market for aluminium scrap, post-consumer | aluminium scrap, post-consumer | APOS, U - GLO	Ecoinvent 3.2 [8]
average incineration residue	−2.0	kg	treatment of average incineration residue, residual material landfill | average incineration residue | APOS, U - CH	Ecoinvent 3.2 [8]
Copper	4.4	kg	treatment of used cable | copper | APOS, U - GLO	Ecoinvent 3.2 [8]
diesel, burned in building machine	41.0	MJ	diesel, burned in building machine | diesel, burned in building machine | APOS, U - GLO	Ecoinvent 3.2 [8]
electricity, medium voltage	113.6	kWh	market for electricity, medium voltage | electricity, medium voltage | APOS, U - IT	Ecoinvent 3.2 [8]
glass cullet, sorted	686.0	kg	market for glass cullet, sorted | glass cullet, sorted | APOS, U - GLO	Ecoinvent 3.2 [8]
lime, hydrated, loose weight	36.5	kg	lime production, hydrated, loose weight | lime, hydrated, loose weight | APOS, U - CH	Ecoinvent 3.2 [8]
limestone residue	−306.1	kg	treatment of limestone residue, inert material landfill | limestone residue | APOS, U - CH	Ecoinvent 3.2 [8]
nitric acid, without water, in 50% solution state	7.1	kg	nitric acid production, product in 50% solution state | nitric acid, without water, in 50% solution state | APOS, U - RER	Ecoinvent 3.2 [8]
silicon carbide	34.7	kg	treatment of spent sawing slurry from Si-wafer cutting | silicon carbide | APOS, U - RER	Ecoinvent 3.2 [8]
sludge, pig iron production	−50.3	kg	treatment of sludge, pig iron production, residual material landfill | sludge, pig iron production | APOS, U - CH	Ecoinvent 3.2 [8]
waste electric wiring	−0.6	kg	treatment of waste electric wiring, collection for final disposal | waste electric wiring | APOS, U - RoW	Ecoinvent 3.2 [8]
waste glass	−14.0	kg	treatment of waste glass, inert material landfill | waste glass | APOS, U - CH	Ecoinvent 3.2 [8]
waste plastic, mixture	−51.0	kg	treatment of waste plastic, mixture, municipal incineration | waste plastic, mixture | APOS, U - CH	Ecoinvent 3.2 [8]
waste polyvinylfluoride	−15.0	kg	treatment of waste polyvinylfluoride, municipal incineration | waste polyvinylfluoride | APOS, U - CH	Ecoinvent 3.2 [8]
Waste treatment PV	1000.0	kg		Reference input
waste wire plastic, municipal incineration	−5.0	kg	treatment of waste wire plastic, municipal incineration | waste wire plastic | APOS, U - CH	Ecoinvent 3.2 [8]
water, completely softened, from decarbonised water, at user |	309.7	kg	water production, completely softened, from decarbonised water, at user | water, completely softened, from decarbonised water, at user | APOS, U - RER	Ecoinvent 3.2 [8]
*Output*				
Nitrogen oxides	2.0	kg		
aluminium scrap, new	182.7	kg	market for aluminium scrap, new | aluminium scrap, new | APOS, U - RER	Avoided product Ecoinvent 3.2 [8]
copper scrap, sorted, pressed	4.4	kg	market for copper scrap, sorted, pressed | copper scrap, sorted, pressed | APOS, U - GLO	Avoided product Ecoinvent 3.2 [8]
electricity, medium voltage	248.8	MJ	electricity voltage transformation from high to medium voltage | electricity, medium voltage | APOS, U - IT	Avoided product Ecoinvent 3.2 [8]
glass cullet	686.0	kg	market for glass cullet, for Saint-Gobain ISOVER SA | glass cullet, for Saint-Gobain ISOVER SA | APOS, U - GLO	Avoided product Ecoinvent 3.2 [8]
heat, district or industrial, natural gas	502.8	MJ	heat production, natural gas, at industrial furnace >100kW | heat, district or industrial, natural gas | APOS, U - Europe without Switzerland	Avoided product Ecoinvent 3.2 [8]
silicon, metallurgical grade	34.7	kg	market for silicon, metallurgical grade | silicon, metallurgical grade | APOS, U - GLO	Avoided product Ecoinvent 3.2 [8]

**Table 16:** Life Cycle Inventory of Inverters and a Charge Controllers (adapted from Inverter) end of life [18].

Component	Amount	Unit	Process	Comments and Sources
Output				
aluminium scrap, post-consumer	−5.0	kg	treatment of aluminium scrap, post-consumer, by collecting, sorting, cleaning, pressing | aluminium scrap, post-consumer | APOS, U - RER	Ecoinvent 3.2 [8]
Copper	1.9	kg	treatment of used cable | copper | APOS, U - GLO	Ecoinvent 3.2 [8]
electronics scrap from control unit	−0.9	kg	treatment of electronics scrap from control units | electronics scrap from control units | APOS, U - RER	Ecoinvent 3.2 [8]
Inverter/charge controller	1.0	Items		Reference input
hazardous waste, for incineration	−12.8	Wh	treatment of hazardous waste, hazardous waste incineration | hazardous waste, for incineration | APOS, U - CH	Ecoinvent 3.2 [8]
iron scrap, sorted, pressed	0.9	kg	sorting and pressing of iron scrap | iron scrap, sorted, pressed | APOS, U - RER	Ecoinvent 3.2 [8]
municipal solid waste	−0.2	kg	treatment of municipal solid waste, municipal incineration with fly ash extraction | municipal solid waste | APOS, U - CH	Ecoinvent 3.2 [8]
used printed wiring boards	−1.2	kg	market for used printed wiring boards | used printed wiring boards | APOS, U - GLO	Ecoinvent 3.2 [8]
waste paperboard	−1.8	kg	treatment of waste paperboard, municipal incineration | waste paperboard | APOS, U - CH	Ecoinvent 3.2 [8]
waste polyethylene	−11.5	g	treatment of waste polyethylene, municipal incineration | waste polyethylene | APOS, U - CH	Ecoinvent 3.2 [8]
wastewater, unpolluted	−19.9	l	treatment of wastewater, unpolluted, capacity 5E9l/year | wastewater, unpolluted | APOS, U - CH	Ecoinvent 3.2 [8]
Output				
aluminium, cast alloy	5.0	kg	market for aluminium, cast alloy | aluminium, cast alloy | APOS, U - GLO	Avoided product Ecoinvent 3.2 [8]
Copper	1.9	kg	market for copper | copper | APOS, U - GLO	Avoided product Ecoinvent 3.2 [8]
iron ore, crude ore, 46% Fe	0.9	kg	market for iron ore, crude ore, 46% Fe | iron ore, crude ore, 46% Fe | APOS, U - GLO	Avoided product Ecoinvent 3.2 [8]

**Table 17:** Life Cycle Inventory of a PEMFCs and PEMEs end of life [19].

Component	Amount	Unit	Process	Comments and Sources
*Input*				
aluminium scrap, post-consumer	−57.5	kg	market for aluminium scrap, post-consumer | aluminium scrap, post-consumer | APOS, U - GLO	Ecoinvent 3.2 [8]
copper	−9.5	kg	treatment of used cable | copper | APOS, U - GLO	Ecoinvent 3.2 [8]
hazardous waste, for incineration	−5.6	kg	treatment of hazardous waste, hazardous waste incineration | hazardous waste, for incineration | APOS, U - CH	Ecoinvent 3.2 [8]
inert waste, for final disposal	−9.8	kg	market for inert waste, for final disposal | inert waste, for final disposal | APOS, U - GLO	Ecoinvent 3.2 [8]
scrap copper	−2.5	kg	market for scrap copper | scrap copper | APOS, U - GLO	Ecoinvent 3.2 [8]
scrap steel	−23.1	kg	treatment of scrap steel, inert material landfill | scrap steel | APOS, U - CH	Ecoinvent 3.2 [8]
slag from metallurgical grade silicon production	−0.2	kg	treatment of slag from metallurgical grade silicon production, inert material landfill | slag from metallurgical grade silicon production | APOS, U - CH	Ecoinvent 3.2 [8]
waste aluminium	−50.0	g	treatment of waste aluminium, sanitary landfill | waste aluminium | APOS, U - CH	Ecoinvent 3.2 [8]
Waste management 3kW FC	1	Items		Reference input
waste plastic, industrial electronics	−22.4	kg	market for waste plastic, industrial electronics | waste plastic, industrial electronics | APOS, U - GLO	Ecoinvent 3.2 [8]
*Output*				
aluminium, cast alloy	58.6	kg	market for aluminium, cast alloy | aluminium, cast alloy | APOS, U - GLO	Avoided product Ecoinvent 3.2 [8]
steel, unalloyed	140.2	kg	market for steel, unalloyed | steel, unalloyed | APOS, U - GLO	Avoided product Ecoinvent 3.2 [8]

**Table 18:** Life Cycle Inventory of platinum recovery process [20] from PEMFCs and PEMEs membranes.

Component	Amount	Unit	Process	Comments and Sources
*Input*				
1-pentanol	620.0	kg	hydroformylation of butene | 1-pentanol | APOS, U - RER	Ecoinvent 3.2 [8]
ammonium chloride	26.6	kg	market for ammonium chloride | ammonium chloride | APOS, U - GLO	Ecoinvent 3.2 [8]
Phosphoryl chloride	36.6	kg	phosphoryl chloride production | phosphoryl chloride | APOS, U - RER	Cyanex production [20]
Solvent, organic	80.4	kg	market for solvent, organic | solvent, organic | APOS, U - GLO	Cyanex production [20]
hazardous waste	−1.4	kg	treatment of hazardous waste, hazardous waste incineration | hazardous waste, for incineration | APOS, U - CH	Ecoinvent 3.2 [8]
hydrochloric acid, without water, in 30% solution state	284.0	kg	tetrafluoroethane production | hydrochloric acid, without water, in 30% solution state | APOS, U - GLO	Ecoinvent 3.2 [8]
hydrogen peroxide, without water, in 50% solution state	5.0	kg	hydrogen peroxide production, product in 50% solution state | hydrogen peroxide, without water, in 50% solution state | APOS, U - RER	Ecoinvent 3.2 [8]
sodium hydroxide, without water, in 50% solution state	74.0	kg	market for sodium hydroxide, without water, in 50% solution state | sodium hydroxide, without water, in 50% solution state | APOS, U - GLO	Ecoinvent 3.2 [8]
spent solvent mixture	737.0	kg	clinker production | spent solvent mixture | APOS, U - CH	Ecoinvent 3.2 [8]
Waste Pt	1.0	kg		Reference input
wastewater, average	−1.9	m^3^	treatment of wastewater, average, capacity 4.7E10l/year | wastewater, average | APOS, U - CH	Ecoinvent 3.2 [8]
water, deionised, from tap water, at user	1900.0	kg	water production, deionised, from tap water, at user | water, deionised, from tap water, at user | APOS, U - CH	Ecoinvent 3.2 [8]
*Output*				
Platinum	0.7	kg	market for platinum | platinum | APOS, U - GLO	Avoided product Ecoinvent 3.2 [8]

**Table 19:** Life Cycle Inventory of carbon fibre recovery process [20] from Hydrogen Storage Tanks.

Component	Amount	Unit	Process	Comments and Sources
*Input*				
acetic acid, without water, in 98% solution state	250.0	g	market for acetic acid, without water, in 98% solution state | acetic acid, without water, in 98% solution state | APOS, U - GLO	Ecoinvent 3.2 [8]
electricity, low voltage	1.0	kWh	market for electricity, low voltage | electricity, low voltage | APOS, U - IT	Ecoinvent 3.2 [8]
polymer foaming	200.0	g	market for polymer foaming | polymer foaming | APOS, U - GLO	Ecoinvent 3.2 [8]
waste carbon fibre	556.0	g		Reference input
sodium hydroxide, without water, in 50% solution state	20.0	g	market for sodium hydroxide, without water, in 50% solution state | sodium hydroxide, without water, in 50% solution state | APOS, U - GLO	Ecoinvent 3.2 [8]
water, deionised, from tap water at user	750.0	g	market for water, deionised, from tap water, at user | water, deionised, from tap water, at user | APOS, U - GLO	Ecoinvent 3.2 [8]
*Output*				
carbon fibre	300.0	g		Avoided product Carbon fibre production [7]

## 2 Experimental design, materials, and methods

Data are represented in Tables divided in two sections: Inputs and Outputs.

• The first column collects the Ecoinvent 3.2 reference flows;
• The second column contains the amount of energy or material whose evaluation is based on the Nano-grid design and modelling as described in Ref. [1]. A negative number must be used in end of life processes because of the logic used by Ecoinvent in building these processes;
• The third column contains the unit of measurement of inputs and outputs;
• The fourth column contains the provider process for the flows;
• The fifth column contains sources and comments. The whole inventory is based on Ecoinvent 3.2 but when a component is not available in the database, information has been gathered from scientific papers in the literature. Based on literature data, the inventory of the missing components has been built using Ecoinvent 3.2 [5]. Other comments specify if the flow represents a reference flow, which means that the provider is the process described it the table itself, or an avoided product to estimate the environmental benefits of recycling processes.

Table 1 represents the inventory for the manufacturing of a tank storing gaseous hydrogen at 350 bar (Type III).

Table 2 represents the inventory for the manufacturing of a tank storing gaseous hydrogen at 700 bar (Type IV).

Table 3 represents the inventory for a Solar Home System equipped with the lithium iron phosphates (LFP) batteries studied by Majeau-Bettez et al. [9] (M-B) whose inventory is provided by Peters and Weil [10].

Table 4 represents the inventory for a Solar Home System equipped with the lithium iron phosphates (LFP) batteries studied by Zackrisson et al. [12] (Zack) whose inventory is provided by Peters and Weil [10].

Table 5 represents the inventory for a Solar Home System equipped with the lithium titanate (LTO) batteries studied by Bauer [13] whose inventory is provided by Peters and Weil [10].

Table 6 represents the inventory for a Solar Home System equipped with the lithium manganese oxide (LMO) batteries studied by Notter et al. [14] whose inventory is provided by Peters and Weil [10].

Table 7 represents the inventory for a Solar Home System equipped with the lithium nickel cobalt aluminium (NCA) oxide batteries studied by Bauer [13] whose inventory is provided by Peters and Weil [10].

Table 8 represents the inventory for a Solar Home System equipped with the lithium nickel cobalt manganese oxide (NCM) batteries studied by Ellingsen et al. [15] (Ell) whose inventory is provided by Peters and Weil [10].

Table 9 represents the inventory for a Solar Home System equipped with the lithium nickel cobalt manganese (NCM) oxide batteries studied by Majeau-Bettez et al. [9] (M-B) whose inventory is provided by Peters and Weil [10].

Table 10 represents the inventory for a hybrid Nano-grid (HNG) equipped with the lithium nickel cobalt aluminium oxide (NCA) batteries studied by Bauer [13] whose inventory is provided by Peters and Weil [10] and with hydrogen storage. In this scenario (A) hydrogen is stored at 350 bar, produced by electrolysers powered by photovoltaics and converted to electricity by fuel cells whose lifespan is supposed to be 12.000 hours.

Table 11 represents the inventory for a hybrid Nano-grid (HNG) equipped with the lithium nickel cobalt aluminium (NCA) oxide batteries studied by Bauer [13] whose inventory is provided by Peters and Weil [10] and with hydrogen storage. In this scenario (B) hydrogen is stored at 700 bar, produced by electrolysers powered by photovoltaics and converted to electricity by fuel cells whose lifespan is supposed to be 12.000 hours.

Table 12 represents the inventory for a hybrid Nano-grid (HNG) equipped with the lithium nickel cobalt aluminium oxide (NCA) batteries studied by Bauer [13] whose inventory is provided by Peters and Weil [10] and with hydrogen storage. In this scenario (C) hydrogen is stored at 350 bar, produced by electrolysers powered by photovoltaics and converted to electricity by fuel cells whose lifespan is supposed to be 60.000 hours.

Table 13 represents the inventory for a hybrid Nano-grid (HNG) equipped with the lithium nickel cobalt aluminium oxide (NCA) batteries studied by Bauer [13] whose inventory is provided by Peters and Weil [10] and with hydrogen storage. In this scenario (B) hydrogen is stored at 700 bar, produced by electrolysers powered by photovoltaics and converted to electricity by fuel cells whose lifespan is supposed to be 60.000 hours.

Table 14 represents the inventory for a generic lithium-ion battery end of life management, where part of the materials is recovered [16].

Table 15 represents the inventory for a crystalline photovoltaic (PV) panel end of life management where part of the materials is recovered [17].

Table 16 represents the inventory for an inverter end of life management where part of the materials is recovered [18]. As no inventory for charge controllers end of life management is available in the literature, this component has been approximated to an inverter as both are electric converters composed of many other small electronic sub-components.

Table 17 represents the inventory for proton exchange membrane electrolysers (PEMEs) and fuel cells (PEMFCs) end of life management, electrochemical devices composed of the same materials that are partially recovered [19].

Table 18 represents the inventory for platinum recovery from PEMEs and PEMFCs membranes as, even if the use of this rare material could be impactful for the environment, it was not considered in Ref. [19].

Table 19: as no inventory exists for hydrogen storage tanks end of life management, a recovering process has been considered for carbon fibre, representing the most weighting material of the tanks.

## Funding

This research did not receive any specific grant from funding agencies in the public, commercial, or not-for-profit sectors.
